# The Influence of Dimensional Parameters on the Characteristics of Magnetic Flux Concentrators Used in Tunneling Magnetoresistance Devices

**DOI:** 10.3390/s25154739

**Published:** 2025-07-31

**Authors:** Ran Bi, Huiquan Zhang, Shi Pan, Xinting Liu, Ruiying Chen, Shilin Wu, Jun Hu

**Affiliations:** Department of Electrical Engineering, Tsinghua University, Beijing 100084, China; br20@mails.tsinghua.edu.cn (R.B.); zhq22@mails.tsinghua.edu.cn (H.Z.); ps21@mails.tsinghua.edu.cn (S.P.); xt-liu22@mails.tsinghua.edu.cn (X.L.); chenry@mail.tsinghua.edu.cn (R.C.); wushilin@tsinghua.edu.cn (S.W.)

**Keywords:** magnetic sensors, magnetic circuit, magnetic flux concentrator, finite element simulation, tunneling magnetoresistance

## Abstract

Measuring weak magnetic fields proposes significant challenges to the sensing capabilities of magnetic field sensors. The magnetic field detection capacity of tunnel magnetoresistance (TMR) sensors is often insufficient for such applications, necessitating targeted optimization strategies to improve their performance in weak-field measurements. Utilizing magnetic flux concentrators (MFCs) offers an effective approach to enhance TMR sensitivity. In this study, the finite element method was employed to analyze the effects of different MFC geometric structures on the uniformity of the magnetic field in the air gap and the magnetic circuit gain (MCG). It was determined that the MCG of the MFC is not directly related to the absolute values of its parameters but rather to their ratios. Simulation analyses evaluated the impact of these parameter ratios on both the MCG and its spatial distribution uniformity, leading to the formulation of MFC design optimization principles. Building on these simulation-derived principles, several MFCs were fabricated using the 1J85 material, and an experimental platform was established to validate the simulation findings. The fabricated MFCs achieved an MCG of 7.325 times. Based on the previously developed TMR devices, a detection sensitivity of 2.46 nT/$\sqrt{\mathrm{Hz}}$ @1Hz was obtained. By optimizing parameter configurations, this work provides theoretical guidance for further enhancing the performance of TMR sensors in magnetic field measurements.

## 1. Introduction

Weak magnetic field measurement plays a crucial role in various fields [1,2,3,4,5,6], particularly influencing the development of geological exploration, deep space exploration, and biomedicine. To measure weak magnetic fields in these areas, multiple magnetic field measurement methods have been explored [7,8,9,10]. Among them, magnetoresistive (MR) effect sensors [11] are promising due to their high sensitivity, compact size, and low cost of mass production. Tunnel junction magnetoresistance (TMR) sensors particularly stand out due to their high sensitivity [12,13]. They are distinct from other magnetoresistive effect sensors, such as anisotropic magnetoresistance (AMR) and giant magnetoresistance (GMR), due to their higher sensitivity. This exceptional sensitivity makes them a key focus of research in the next generation of magnetic field sensors.

Although TMR has higher sensitivity, its detection capability is still limited to the tens of nT level. Achieving detection capabilities at the nT and pT levels in the field of weak magnetic field measurement remains a major challenge for TMR sensors. Research aimed at improving the sensitivity of TMR sensors remains an active and extensive field of study.

The enhancement of TMR sensor performance basically involves two approaches. One approach focuses on optimizing the parameters of TMR sensors, such as the materials and geometric dimensions of multilayers, as well as optimizing fabrication processes, with the aim of enhancing TMR sensitivity and reducing noise. For example, some research groups have analyzed Heusler alloys to enhance the spin polarization of electrons in TMR sensors, thereby increasing the magnetoresistance to $3.43\times 10^{7}\%$ [14]. However, Heusler alloys face challenges related to thermal instability and lattice mismatch issues, which currently hinder their applications [15]. Other research groups introduced materials such as CoFeSiB [16] and CoFeBTa [17] to enhance the soft magnetic properties of the free layer in TMR sensors, thereby increasing sensitivity. Additionally, studies by Jin Zhenhu et al. [18] investigated the relationship between sensors geometric dimensions and TMR sensitivity using rectangular cross-section configurations. Our research team [19] has also explored elliptical cross-section TMR structures, successfully developing sensors capable of nT-level detection sensitivity in the previous research.

The second approach to enhancing TMR performance is based on optimizing the magnetic circuit of the TMR sensors. Specifically, this is typically achieved by employing magnetic flux concentrators (MFCs) to amplify the magnetic field detected by the TMR sensors. These MFCs are usually fabricated from magnetic material with high permeability and can improve the minimum detectable field in biomedical applications without compromising sensor performance [20,21]. Micro-scale MFC designs have been employed in research aimed at magnetic field amplification [22]. Other research on MFCs, such as Kanno, A. [23], enhanced the performance of TMR sensors using MFCs by 10 times to measure brain magnetic signals at the scalp and observed brain magnetic signals induced by neural stimulation. Marília Silva [24] and Guanyang He [25] combined integrated MFCs with macro-scale MFCs, resulting in sensitivity enhancements of 464-fold and 517-fold, respectively. Guanyang He utilized NiFeCuMo as the material, whereas Marília Silva employed a bilayer structure consisting of NiFe and CoZrNb. Related studies on macro-scale MFCs include Xiaoming Zhang [26] and Hariharan Nhalil [27], who used finite element method simulations to analyze the relationship between the magnetic circuit gain (MCG) of MFCs and parameters of MFCs, guiding the fabrication of corresponding MFCs with enhanced magnetic field amplification. In Shih-Jui Chen’s research [28], three-dimensional (3D) MFCs were utilized to amplify magnetic fields, enabling their application in magnetometers for measuring weak 3D magnetic signals. Yi Yang [29] investigated the influence of multi-layer structures on the coercive force of MFCs, while X. Sun [30] investigated how the geometric configuration and material characteristics influence the magnetic amplification achieved by MFCs.

In practical applications, it is essential to determine the geometric size parameters of MFCs based on design principles to achieve optimal MCG. This study employed finite element simulation to establish design rules for MFCs and fabricated them using 1J85 permalloy. In the simulation process, COMSOL Multiphysics (version 6.3) was employed to analyze the influence of different geometric parameters on the MCG, focusing on the effects of parameters maintained at the same ratio but with varying absolute values. The results revealed that the MCG depends solely on the proportional relationships among parameters, independent of their absolute values. Subsequently, the effects of various geometric parameters on the uniformity of MCG across the air gap were simulated. Then, the influence of parameter ratios on MCG was analyzed, reaching simplified fitting models and the formulation of design guidelines for MFCs. In the experimental phase, MFCs with different shapes were fabricated and applied into a testing platform to validate their MCG. Based on previous work and MFCs fabrication, a TMR sensor with a detection at the 2.46 nT level was realized.

## 2. Theoretical Analysis and Simulation

### 2.1. Magnetic Circuit Theory Analysis

**Figure 1:**
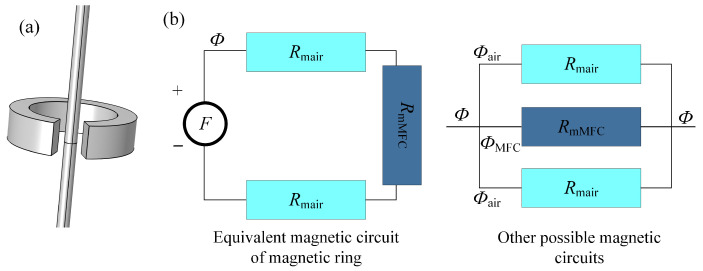
(**a**) Magnetic ring structure, (**b**) magnetic circuit equivalent model.

For MFCs, which typically consist of high magnetic permeability materials combined with air gaps, two primary theoretical approaches are commonly employed to analyze their parameters: one is based on magnetic circuit theory, and the other is based on field theory (Maxwell’s equations combined with finite element methods). These two methods can play a significant role in the parameter design of MFCs across different structural configurations. In closed magnetic circuits, the MCG of the MFC can be derived using simplified magnetic circuit analysis. Conversely, in open or non-closed circuits, field theory approaches are necessary for accurate analysis. To better understand the mechanism by which the MFC amplifies the magnetic induction, we initially adopt magnetic circuit theory to analyze a simple MFC structure, as illustrated in Figure 1a.

In a magnetic field, the relationship between magnetic flux and magnetic field can be expressed as (1).

(1) $$\Phi ={\;\int \int \;}_{S}\vec{B}\cdot d\vec{A}$$

where Φ is the magnetic flux, *B* is the magnetic induction, and *A* is the area vector. When considering a surface perpendicular to the magnetic field as the area element for calculating flux, the directions of *A* and *B* are aligned. In this case, Equation (1) can be simplified to (2).

(2) $$\Phi =BS=\mu HS=\mu \frac{F}{l}S$$

where *H* represents the magnetic field intensity, *F* denotes the magnetomotive force, μ is the permeability of the material, and *l* is the length of the magnetic circuit.

The simple magnetic circuit theory is shown in Figure 1b. Magnetic circuit theory shares similarities with electrical circuit theory, where *F* acts as the driving force of the magnetic field, analogous to voltage in electrical circuits. The magnetic reluctance ($R_{m}$), which opposes the magnetic flux passing through the magnetic circuit, is similar to electrical resistance. The $R_{m}$ can be expressed as $R_{m}=l/\left(\mu S\right)$. Magnetic flux (Φ) represents the total flux density passing through the magnetic circuit, akin to electric current in electrical circuits.

When the geometry of the MFC is uniform, the MCG can be conveniently calculated using analytical methods. For example, the magnetic ring in Figure 1a, which is commonly used in current measurement applications, is also a regular MFC. Under such conditions, the MCG can be expressed as (3) [31].

(3) $$\begin{array}{cc} \; & B_{1}=\frac{2\mu_{0}I}{\pi (D_{1}+D_{2})}\\ \; & B_{2}=\frac{\mu_{0}\mu_{r}I}{\mu_{r}d+\pi \left(D_{1}+D_{2}\right)/2-d}\\ \; & k=\frac{B_{2}}{B_{1}}=\frac{\mu_{r}\pi \left(D_{1}+D_{2}\right)}{2\mu_{r}d+\pi \left(D_{1}+D_{2}\right)-2d} \end{array}$$

where $B_{1}$ represents the magnetic induction when no MFC is present, while $B_{2}$ denotes the magnetic induction after the MFC is applied. $D_{1}$ and $D_{2}$ are the diameters of the inner and outer diameter of the magnetic ring, respectively. The parameters $\mu_{0}$ and $\mu_{r}$ are vacuum permeability and the relative permeability of the magnetic ring. The parameter *d* is the air gap length of the magnetic ring. The parameter *I* is the current in the conductor. The parameter *k* corresponds to the MCG. The MCG in the article refers to the ratio of the magnetic induction at the same location before and after using the MFC. In other words, it represents the amplification factor of the magnetic induction in the air gap resulting from the application of the MFC.

The calculation of the MCG uses the circular symmetry of the magnetic field generated by a long straight conductor. Simultaneously, it also incorporates the continuity of the magnetic induction (*B*) normal to the material interface, ensuring a correct analysis of MCG.

However, the application scope of MFCs with type of magnetic rings is significantly limited, primarily confined to the measurement of long conductors. For MFCs with irregular geometries that prevent direct calculation of magnetic reluctance, or in cases of non-closed magnetic circuits, simple analytical methods based on magnetic circuit theory are insufficient to determine the MCG. In such situations, field theory approaches are necessary to accurately obtain the MCG of the MFC.

### 2.2. Simulation Setup

**Figure 2:**
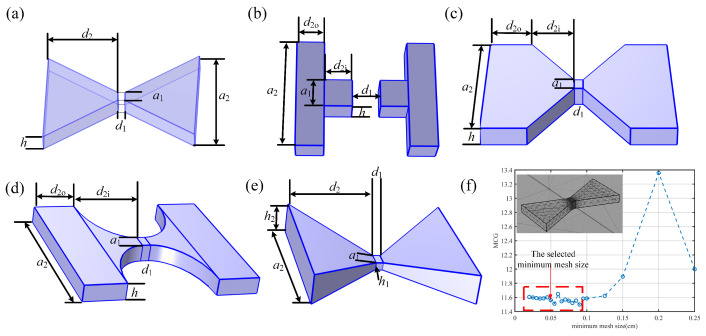
(**a**) Trapezoidal-shaped MFC structure, (**b**) T-shaped MFC structure, (**c**) triangular-shaped MFC structure, (**d**) trumpet-shaped MFC structure, (**e**) wedge-shaped MFC structure, (**f**) the effect of mesh size on MCG calculation simulation result.

Based on the review of research on MFCs, the geometrical configurations typically include several structures, as illustrated in Figure 2. These consist of trapezoidal-shaped (Figure 2a), T-shaped (Figure 2b), triangular-shaped (Figure 2c), trumpet-shaped (Figure 2d), and wedge-shaped (Figure 2e) structures. The parameters of each MFC are annotated in Figure 2, with common parameters shared by all MFCs being thickness (*h*), air gap length ($d_{1}$), MFC length ($d_{2}$) (longitudinal parameters), air gap width ($a_{1}$), and MFC width ($a_{2}$) (transverse parameters). For the T-shaped, triangular-shaped, and trumpet-shaped structures, the length ($d_{2}$) is subdivided into ($d_{2}i$) and ($d_{2}o$), representing the inner and outer lengths, respectively. In the wedge-shaped MFC, the thickness (*h*) is divided into ($h_{1}$) and ($h_{2}$), corresponding to the inner and outer thicknesses.

In COMSOL Multiphysics, the magnetic field (mf) physics interface was utilized for simulation. Considering that the material used for fabricating the MFCs is 1J85 permalloy, which has a typical relative magnetic permeability ranging from 2000 to 8000, the relative permeability was set to a value of 4000 for the simulations. The relative magnetic permeability of air was configured as 1. Since two different materials are present in the domain, two separate magnetic flux conservation equations must be applied accordingly. The built-in interface within COMSOL also incorporates magnetic boundary conditions. The governing equations mentioned above are, respectively, represented by Equations (4) and (5).

In the boundary conditions, $\vec{M}$ denotes the magnetization vector, together with *B* and *H* representing the boundary conditions at interfaces between different materials, respectively. Since the magnetic field is a source-free field, the divergence of the magnetic induction (5) is zero. Utilizing the divergence relationship between the current density *J*, the time derivative of the charge *D*, and magnetic field intensity, comprehensive boundary and initial conditions can be established for accurate simulation. Once the initial magnetic field in the simulation domain is specified, COMSOL’s numerical methods are employed to compute the magnetic field distribution enhanced by the MFC, reaching results of MCG.

(4) $$\begin{array}{cc} \; & \vec{B}=\mu_{0}\mu_{r}\left(\vec{H}+\vec{M}\right)\\ \; & \vec{B}=\mu_{0}\vec{H} \end{array}$$

(5) $$\begin{array}{cc} \; & \nabla \cdot \vec{B}=0\\ \; & \nabla \times H=J+\frac{\partial D}{\partial t} \end{array}$$

To enhance the accuracy of the MCG calculations and improve the computational efficiency of the COMSOL simulations, special meshing strategies were implemented. The overall meshing approach employed sweeping and mapping methods, with the simulation domain divided into four distinct mesh regions, each tailored to its specific area. The outermost region, representing the air domain, was modeled using an infinite element boundary to simulate an infinitely distant space, thereby increasing the precision of the results. The middle layer of the air domain was meshed with a regular mesh size governed by the physical field control settings.

The mesh sizes in the MFC regions and the air gaps between MFC structures were set proportionally to the geometrical parameters of the MFC, ensuring that each dimension of the simulation domain contained at least 3 to 5 mesh layers. This meshing strategy was adopted to optimize the fidelity of the MCG analysis within COMSOL, as shown in Figure 2f. We conducted a simulation analysis of the influence of mesh size on the calculation of MCG, with the minimum mesh size located in the air gap where $d_{1}$ = 1 cm. When the minimum mesh size is less than 0.1 cm, further reducing the mesh size results in the MCG variation staying within ±0.3%. We selected the more stable mesh size of 0.05 cm as the minimum mesh size. At this size, the smallest mesh in the air gap is $d_{1}$/20, and the largest is $d_{1}$/5. In the MFC region, the minimum mesh size was set to $a_{1}/20\times 3$ and the maximum to $a_{1}/4\times 3$, accommodating the inner and outer dimensions (note that $a_{2}$ is typically larger than 5 times $a_{1}$ in simulation).

### 2.3. COMSOL Simulation Analysis Results

**Figure 3:**
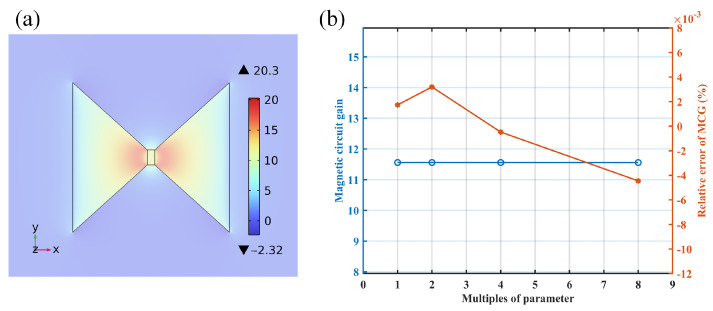
(**a**) MCG distribution of the MFC; (**b**) MCG and relative error of MCG with various multiple of parameters.

Initially, simulations were conducted on trapezoidal MFCs, which have fewer parameters. This initial step was then extended to the MFCs with other geometric configurations. To verify that the parameters being analyzed comprehensively determine the MCG performance of the MFCs, simulations in COMSOL were performed using the parameters with proportional values. Using base parameters of $d_{1}$ = 1 cm, $d_{2}$ = 10 cm, $a_{1}$ = 1 cm, $a_{2}$ = 10 cm, and *h* = 2 cm, the simulations were conducted with these parameters scaled up to 2, 4, and 8 times their initial values. The results obtained are illustrated in the accompanying Figure 3.

When all structural parameters change proportionally, the ratio of the magnetic induction at the center point of the MFC’s air gap to the applied uniform field remains constant (Figure 3b). The relative deviation of the MCG at the MFC air gap center is less than 0.1%. It confirmed that in simulations, the MCG of the MFC is influenced solely by the relative values of the parameters and is independent of their absolute values. This also indicates that no other parameters of MFCs affect the MCG, as all relevant parameters have been considered.

Moreover, since the MCG of the MFC is unaffected by the absolute values of its parameters, the results from macro-scale simulations and experiments can be extrapolated to the design of microscale fabrication schemes. This provides valuable insights for the subsequent development of integrated MFCs.

To facilitate description, we define the direction perpendicular to edges $a_{1}$ and $a_{2}$ as the simulation x-axis, the direction parallel to $a_{1}$ and $a_{2}$ as the simulation y-axis, and the direction parallel to *h* as the z-axis (as also indicated in Figure 3a). In previous work [19], the designed TMR sensor’s cell size is 1.6 mm × 0.8 mm, with an ideal packaging size of 1.6 mm × 1.6 mm for two connected cells. The thickness of this packaging is almost negligible. Therefore, the uniformity of MCG in the air gap is mainly considered along the x-axis and y-axis.

**Figure 4:**
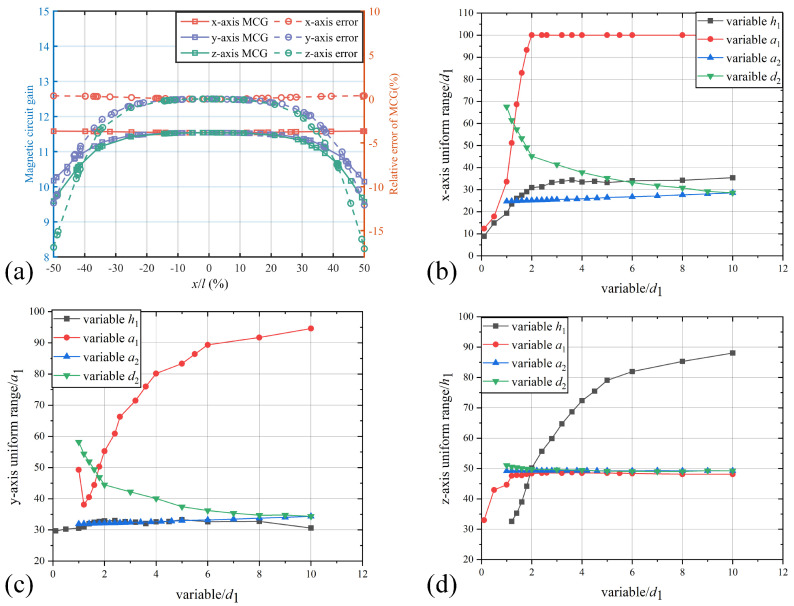
(**a**) Relationship between MCG distribution and x-axis, y-axis, z-axis position; for the x-axis, *l* represents $d_{1}$; for the y-axis, *l* represents $a_{1}$; and for z-axis, *l* represents *h*. Relationship between each parameter and the uniformity along the (**b**) x-axis; (**c**) y-axis; (**d**) z-axis.

The distribution of the MCG and its uniformity along the x-, y-, and z-axes are illustrated in the Figure 4. As shown in Figure 4a, when the MFC parameters are fixed, the MCG significantly decreases near the edges along the y- and z-axes, while it slightly increases near the edges of MFC in the x-axis direction. The dashed lines indicate the relative deviation of the MCG compared to that at the air gap center point, corresponding to the right-side ordinate. The label *x* indicates the position along each axis, while *l* represents the length corresponding to the three axes. For the x-axis, *l* represents $d_{1}$; for the y-axis, *l* represents $a_{1}$; and for the z-axis, *l* represents *h*. Point 0 on the x-axis is located at the midpoint of each *l*.

The impact of parameter ratios on the uniformity of the MCG in the air gap was analyzed. With the air gap length ($d_{1}$) held constant, the analysis includes $d_{2}/d_{1}$, $h/d_{1}$, $a_{1}/d_{1}$, and $a_{2}/d_{1}$, which can determine the MFC performance on uniformity. Similarly, based on the default parameters, $d_{1}$ = 1 cm, $d_{2}$ = 10 cm, $a_{1}$ = 1 cm, $a_{2}$ = 10 cm, and *h* = 2 cm. In simulation, when one parameter varies, the others are still default parameters. Additionally, we consistently set $d_{1}$ = 1 cm and kept it unchanged. Using a standard of 1% deviation of the MCG relative to the center point, the widths that maintain this level of uniformity along each axis were calculated as percentages of the total lengths. These results are displayed as the MCG uniformity outcomes in Figure 4.

From Figure 4b,c, increasing the ratio of the air gap width to the length ($a_{1}/d_{1}$) markedly improves the uniformity of MCG along both the x- and y-axes, with the x-axis uniformity reaching 100% when $a_{1}/d_{1}>2$. When varying the ratio $a_{2}/a_{1}$, the uniformity along the y-axis experiences a slight improvement. In contrast, increasing the ratio $d_{2}/d_{1}$ has a negative effect on the x- and y-axes uniformity of MCG. However, the negative impact of the ratio $d_{2}/d_{1}$ on the uniformity along the x- and y-axes can be readily compensated by increasing $a_{1}/d_{1}$. Since the red curves are all based on parameters with $d_{2}/d_{1}=10$, at this ratio of $d_{2}/d_{1}$, the detrimental effect of ratio on the x- and y-axis uniformities tends to stabilize.

Furthermore, from Figure 4d, it can be observed that the thickness parameter *h* of the MFC significantly influences the uniformity along the z-axis. When the ratio $h/d_{1}$ exceeds 2, the uniform magnetic field gain region within the air gap extends beyond half of the thickness, while the other parameters have negligible influence on the uniformity along the z-axis (Figure 4d). Given that the TMR sensor thickness is typically on the micrometer level, which is negligible relative to the MFC thickness, there is no requirement for an extensive uniform magnetic field region along the z-axis. Therefore, the thickness of the MFC does not need to be excessively large.

Based on the comprehensive analysis, it is evident that optimizing uniformity of MCG along the x- and y-axes within the air gap is critical in the design of MFCs. Consequently, for trapezoidal MFC structures, particular attention should be given to the ratio $a_{1}/d_{1}$ and its compatibility with the parameters of the TMR sensor. Proper coordination of these geometric parameters is essential to achieve optimal magnetic field distribution and sensor performance.

**Figure 5:**
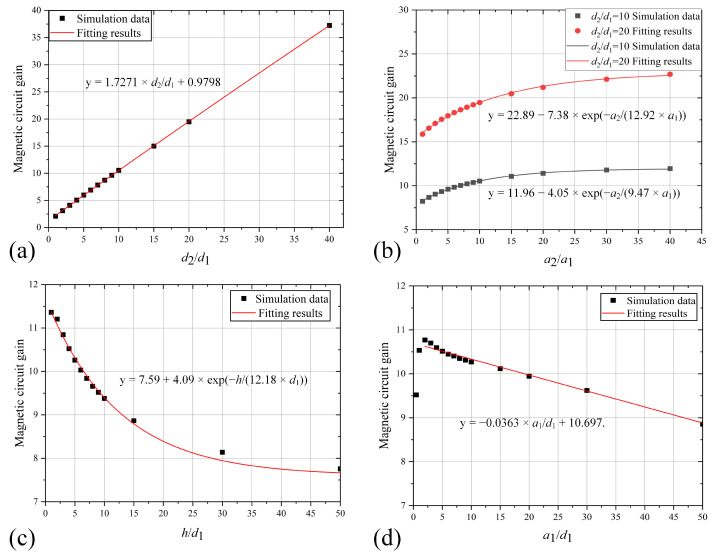
Relationship between each parameter and MCG of trapezoidal MFC (**a**) $d_{2}/d_{1}$; (**b**) $a_{2}/a_{1}$; (**c**) $h_{2}/d_{1}$; (**d**) $a_{1}/d_{1}$.

In the design of the MFC, a key parameter is the value of MCG within the air gap. The relationship between the MCG and the ratios of the four parameter ratios is illustrated in Figure 5.

As shown in Figure 5a, the fitting results between the MCG and the ratio $d_{2}/d_{1}$ indicate that when other parameters remain constant and only $d_{2}$ varies, the MCG of the trapezoidal MFC exhibits an approximately linear relationship with $d_{2}/d_{1}$. Applying a linear fit yields the equation $y=1.7271\times d_{2}/d_{1}+0.9798$, with a residual of $r^{2}=0.99909$. This demonstrates that the MCG of the MFC exhibits a positive correlation with the ratio $d_{2}/d_{1}$.

According to the data presented in Figure 5b, when maintaining the MFC length $d_{2}$ constant, increasing the ratio of the long edge to the narrow edge $a_{2}/a_{1}$ yields only a limited improvement in the MCG. The relationship between the gain and $a_{2}/a_{1}$ was effectively modeled using an exponential fitting function. Specifically, for $d_{2}/d_{1}=10$, the fitted expression is $y=11.96-4.05\times \exp(-a_{2}/\left(9.47\times a_{1}\right))$, and for $d_{2}/d_{1}=20$: $y=22.89-7.38\times \exp(-a_{2}/\left(12.92\times a_{1}\right))$. The corresponding goodness-of-fit metrics, with residuals of 0.9985 and 0.9975, respectively, indicate that these models are effective.

The effect of the ratio between *h* and $d_{1}$ on the MCG is illustrated in Figure 5c. An increase in $h/d_{1}$ results in a decrease in the MCG. This decreasing trend can be effectively modeled using an exponential function, with the fit described by the equation $y=7.59+4.09\times \exp(-h/\left(12.18\times d_{1}\right))$, which yields a residual $r^{2}=0.992$.

When examining the effect of $a_{1}/d_{1}$, the parameter $a_{2}/a_{1}$ was held constant to eliminate its influence. As shown in Figure 5d, increasing $a_{1}/d_{1}$ initially causes the MCG to increase, followed by a decrease. The decreasing segment was fitted using a linear model, yielding the relationship $y=-0.0363\times a_{1}/d_{1}+10.697$. Given that $a_{1}/d_{1}$ typically varies within a narrow range and the coefficient is minor, its impact on the MCG can be approximated as negligible. Consequently, only the influence of $a_{1}/d_{1}$ on the MCG uniformity needs to be considered in the design process.

**Figure 6:**
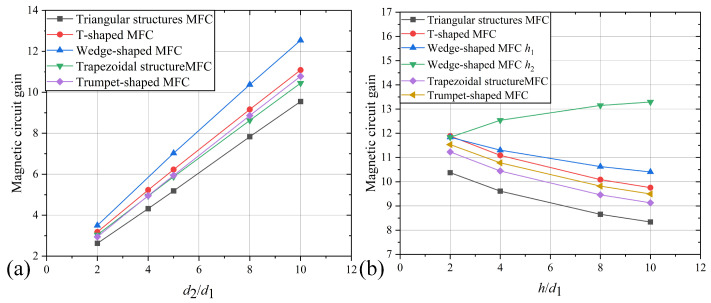
Relationship between parameters and MCG in various structure of MFC. (**a**) $d_{2}/d_{1}$; (**b**) $h/d_{1}$.

Furthermore, analyzing the relationship between the MCG and geometric parameters for other MFC types, the results are shown in Figure 6. It indicates that, similar to the trapezoidal structure, increasing the ratio $d_{2}/d_{1}$ (for T-shaped, trumpet-shaped, and triangular structures, where $d_{2}=d_{2i}+d_{2o}$) results in a linear increase in the MCG.

As depicted in Figure 6b, the MCG for all MFC types exhibits a monotonically decreasing trend with increasing *h*. The green curve illustrates the relationship between the ratio of the external thickness $h_{2}/d_{1}$ and the MCG for a wedge-shaped MFC. When the internal thickness $h/d_{1}$ is held constant, increasing $h_{2}/d_{1}$ can enhance the MCG. However, the percentage increase is approximately $1.5\%/(h_{2}/h_{1})$, indicating that increasing $h_{2}/d_{1}$ alone offers limited benefits for optimizing the MCG of wedge-shaped MFCs, just like increasing $a_{2}/a_{1}$.

**Figure 7:**
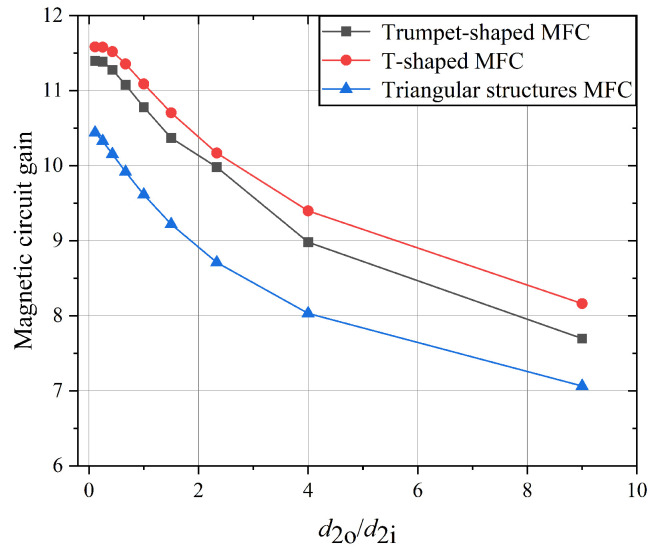
Relationship between $d_{2o}/d_{2i}$ and MCG.

The effects of the internal ratio of $d_{2o}/d_{2i}$ on the optimization of T-shaped, triangular, and trumpet-shaped MFCs were investigated, yielding the simulation results shown in Figure 7. When the combined ratio $\left(d_{2o}+d_{2i}\right)/d_{1}$ is fixed at 10, the MCG decreases as $d_{2o}/d_{2i}$ increases. Specifically, for the triangular MFC, as $d_{2o}/d_{2i}$ approaches zero, the shape approaches that of a trapezoidal MFC, and the MCG converges toward approximately 10.455, which is the MCG value of the trapezoidal MFC under these parameters.

In structures with $d_{2o}$ and $d_{2i}$, the presence of an external rectangular component ($d_{2o}$) exerts a negative influence on the MCG and requires additional geometric space. Therefore, in the design of MFCs, it is advisable to minimize the use of such rectangular structures to optimize magnetic performance and spatial efficiency.

Under identical parameter conditions, the wedge-shaped structure exhibits the highest MCG among the configurations. The T-shaped, trumpet-shaped and trapezoidal MFCs demonstrate comparable MCGs, approximately 10% higher than the triangle structure, indicating an advantage in magnetic performance.

Considering practical aspects such as ease of installation and fabrication, the trapezoidal structure offers the greatest convenience for applications, and fabrication of T-shaped and triangular MFCs is comparatively simpler. Therefore, in Section 3, we focus on the fabrication of trapezoidal, T-shaped, and triangular MFCs, followed by experimental procedures aimed at optimizing the signal-to-noise ratio (SNR) of the TMR sensors.

## 3. Experimental Methods and Discussion

### 3.1. Experimental Platform Construction and Experimental Design

**Figure 8:**
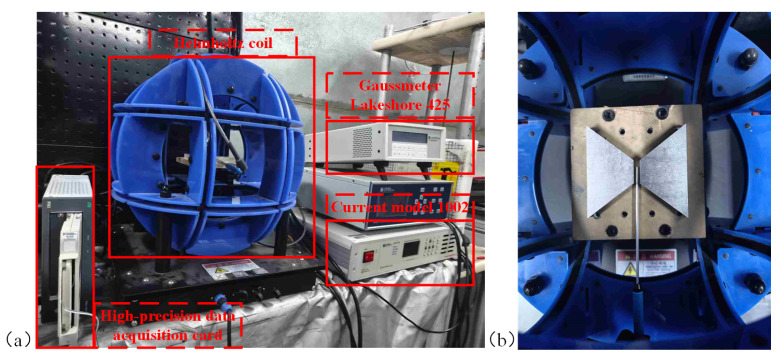
(**a**) Magnetic field testing environment; (**b**) MCG measurement installation schematic.

To evaluate the influence of the MFC structure on the MCG under a magnetic field environment, we constructed a test setup based on Helmholtz coils, as illustrated in Figure 8. The Helmholtz coils generate a uniform magnetic field within a spatial range of 10 × 10 × 10 cm, which covers the operational range of the MFC. The magnetic field produced by the Helmholtz coils is driven by the current source module 1002 (manufactured by Beijing Cuihai Jiacheng Magnetoelectric Technology Co., Ltd., Beijing, China), with a current step of 0.01 mA. The magnetic field-to-current conversion rate of the Helmholtz coils is approximately 10.43 Oe/A, so the minimum magnetic field adjustment step is about $10^{-4}$ Oe (10 nT). The experimental procedure is divided into two steps: the first aims to verify the relationship between the MCG of the MFC and various geometrical parameters. The magnetic field in the air gap was measured using a Lakeshore Gaussmeter 425, which has a minimum resolution of $10^{-3}$ Oe. Although the resolution of the gaussmeter is insufficient to measure the minimal step-sized magnetic field of the Helmholtz coil, such small fields are not necessary when measuring the MCG of the MFC. For the data acquisition system shown in Figure 8a, we used the NI 4309 data acquisition card with background noise that can reach 1–10 nV. This card measures the output voltage of the TMR sensor, and the voltage noise spectral density is obtained through FFT analysis. The NF5307 differential amplifier (background noise can reach 4 nV $\sqrt{\mathrm{Hz}}@$1 Hz), from Beijing Oriental Jicheng Co., Ltd. (Beijing, China), was used to amplify the differential output voltage of the TMR sensor.

Furthermore, by utilizing the MFC to enhance TMR sensitivity, the experiment aims to verify whether this approach can improve the capability for stronger magnetic field detection.

### 3.2. Experimental Methods

**Figure 9:**
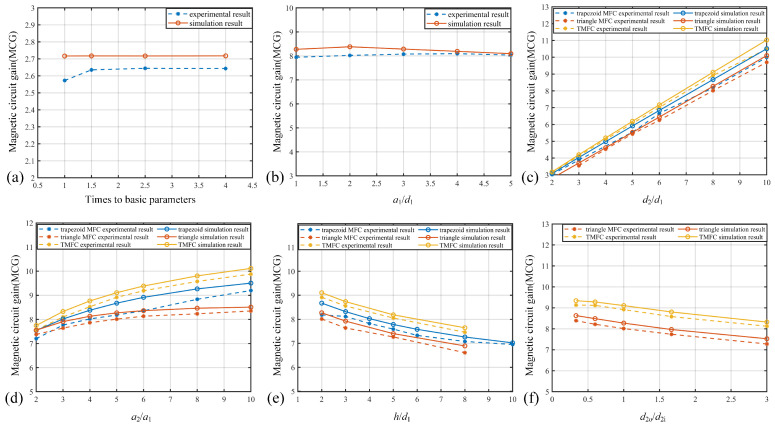
The MCG of MFCs measurement results. (**a**) When the size parameters are scaled proportionally, the trend of the MCG varies accordingly with the scaling factor; (**b**) the effect of the ratio of air gap width ($a_{1}$) to air gap length ($d_{1}$) on the MCG; (**c**) the influence of the ratio of MFC length ($d_{2}$) to the air gap length ($d_{1}$) on MCG; (**d**) the impact of ratio of the outer length ($a_{2}$) to the air gap width ($a_{1}$) on MCG; (**e**) the effect of the ratio of MFC thickness (*h*) to the air gap length ($d_{1}$) on MCG; (**f**) the influence of the ratio $d_{2}o/d_{2}i$ in triangular and T-shaped MFC structures on MCG.

In each subfigure of Figure 9, the solid lines represent the simulation results, while the dashed lines correspond to the experimental measurements. Both sets of results are based on identical parameter configurations. First, the parameter ratios of the MFC were validated and analyzed. Based on the foundational parameters ($d_{1}$ = 0.5 cm, $d_{2}$ = 1 cm, $a_{1}$ = 1 cm, $a_{2}$ = 2 cm, and *h* = 1 cm), MFCs were fabricated with these parameters simultaneously scaled up to 1.5, 2, 2.5, and 4 times their original sizes. The MCG of each MFC was measured, and the results are shown in Figure 9a. When the MFC uses the base parameters, due to the relatively small absolute parameter values, positional errors in placement significantly affect the air gap MCG. As the parameters increase, the influence of placement errors diminishes, and the MCG tends to stabilize. From Figure 9a, it can be inferred that the MCG of the MFC depends solely on the ratio between parameters, rather than their absolute values, which aligns with the simulation results.

Considering that the parameter $a_{1}/d_{1}$ has minimal influence during simulation, only the trapezoidal MFC structure with varying $a_{1}/d_{1}$ was fabricated. The experimental results (in Figure 9b) further indicate that the MCG remains almost unchanged with variations in the $a_{1}/d_{1}$ parameter. Moreover, compared to the simulation outcomes, the impact of $a_{1}/d_{1}$ on the MCG in the experimental data is even less significant. Based on these findings, it can be inferred that the transverse parameters ($a_{1}$, $a_{2}$) and the longitudinal parameters ($d_{1}$, $d_{2}$) are approximately decoupled in their effects on the MCG. That is, the transverse and longitudinal parameters influence the MFC’s MCG independently, and only their individual ratios ($d_{2}/d_{1}$, $a_{2}/a_{1}$, and $h/d_{1}$) need to be considered.

Further tests were conducted to examine the relationship between the MCG of MFCs and various geometrical parameters, with the results shown in Figure 9c. When varying $d_{2}/d_{1}$ (with $a_{2}/a_{1}$ = 5 and $h/d_{1}$ = 2), the MCGs of the three MFC shapes all exhibited a linear increasing trend relative to $d_{2}/d_{1}$. The fitted function is approximately $y=1.014\times d_{2}/d_{1}-0.176$.

As shown in Figure 9d, when changing $a_{2}/a_{1}$ (with $d_{2}/d_{1}$ = 8 and $h/d_{1}$ = 2), the experimental data exhibited a trend similar to exponential growth, aligning with simulation results. The MCG increased with $a_{2}/a_{1}$ and gradually tapered off. The fitted function was $y=10.28-3.81\times exp(-\left(a_{2}/a_{1}\right)/8.17)$, with a residual of 0.9839, indicating good fit accuracy; the functional form matches that observed in simulations.

In Figure 9e, as $h/d_{1}$ increased, the MCG showed a decreasing trend, approximately following an exponential decay pattern. These results collectively demonstrate that the measured relationship between the shape parameters and MCG aligns well with theoretical predictions.

Further validation was conducted to assess the influence of parameters in a two-layer MFC structure. As shown in Figure 9f, increasing the ratio $d_{2o}/d_{2i}$ results in a corresponding decrease in the MCGs.

The MCG values of each MFC in experiment are smaller when compared to the simulation curves, which may be attributed to (1) the air gap during placement being larger than expected, and (2) during heat treatment, the relative magnetic permeability of the MFC may have been lower than the preset value of 4000.

Based on the experimental results, MFCs fabricated using 1J85 material through wire cutting and heat treatment can achieve desirable MCG capabilities. The relationship between the gain and parameter ratios observed experimentally is generally consistent with the simulation outcomes. Therefore, it can be reasonably concluded that simulations provide a reliable prediction of the MFC’s MCG performance.

**Figure 10:**
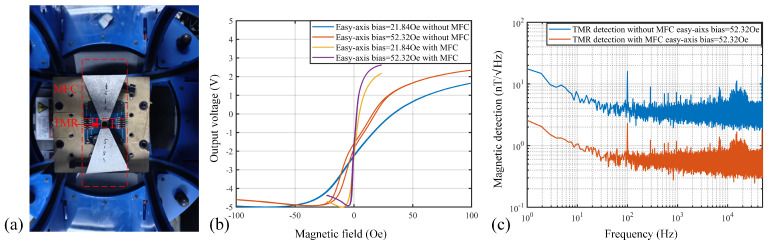
MFC combined with TMR test results. (**a**) Measurement environment with TMR; (**b**) TMR output curve results; (**c**) TMR detection capability curves with and without MFC.

Further, the MFCs were arranged on either side of the TMR device as shown in Figure 10a, and sensitivity and noise measurements were conducted. The TMR device was fabricated previously [19] with a Wheatstone bridge structure. During sensitivity testing, a 1V voltage was applied. The differential outputs of the TMR device were amplified ten-fold using a differential amplifier to obtain the output curve. The results in Figure 10b indicate that incorporating the MFC significantly enhances the TMR sensitivity. Since the TMR package size exceeds 0.5 cm, approximately 0.7 cm, the ratio $d_{2}/d_{1}$ decreases, and although the MCG does not reach the theoretical value of about 10 when $d_{2}/d_{1}\approx 10$, it still provides an approximately seven-fold (7.325-fold) increase in sensitivity. Noise testing was conducted under the same easy-axis bias, 52.32Oe, as in reference [19]. Under these conditions, the TMR’s magnetic field detection capability is around $2.46\mathrm{nT}/\sqrt{\mathrm{Hz}}$ @ 1 Hz. When employing smaller DFN8 packages for the TMR encapsulation, about 2.5-fold sensitivity gain could be achieved through this configuration, thereby enabling more robust magnetic field detection capabilities, about $1\mathrm{nT}/\sqrt{\mathrm{Hz}}$.

## 4. Conclusions and Discussion

This study combined finite element simulations and experimental methods to investigate the influencing factors of MFC magnetic circuit gain (MCG). By comparing the effects of different shapes and size ratios on the MCG, the results elucidate the characteristics of shape-dependent MCG under proportional parameter variations. Through simulation and experimental verification, it was demonstrated that the MCG of the MFC remains unchanged when its absolute size parameters are scaled proportionally; thus, the gain depends solely on the ratios of the parameters rather than their absolute values. This finding supports the extension of such principles to the fabrication of integrated MFCs, serving as a foundational step for subsequent integrated MFC design.

Furthermore, the simulations and experiments confirmed that variations in ratio of air gap length and air gap width have negligible effects on the MCG, effectively decoupling the longitudinal and transverse size influences. Analysis of the combined results revealed that the MCG is positively correlated with the MFC length-to-air gap length ratio. Moreover, the MCG decreases with increasing MFC thickness-to-air gap length ratio and tends to stabilize, while it increases with increasing MFC outer width-to-air gap width ratio and also approaches a stable value. When used in conjunction with TMR sensors, this approach enables an increase in sensitivity without degrading noise performance, thereby improving the magnetic field detection capability.

Building on the TMR fabrication and packaging methods discussed earlier, measurements conducted in low-noise environments achieved a magnetic field detection sensitivity of 2.46 nT/$\sqrt{\mathrm{Hz}}$. Future improvements in packaging—specifically by increasing the MFC length-to-air gap length ratio—are expected to further enhance the MCG, leading to even higher magnetic field detection capabilities.

## Data Availability

Data are contained within the article.
